# Antiretroviral-induced adverse drug reactions in HIV-infected patients in Mali: a resource-limited setting experience

**DOI:** 10.18203/2319-2003.ijbcp20191565

**Published:** 2019-05

**Authors:** Aboubacar Alassane Oumar, Mamadou Dakouo, Anicet Tchibozo, Mamoudou Maiga, Guida Landouré, Raysso Abdi-Bogoreh, Paul M. Tulkens, Sounkalo Dao, Jean Cyr Yombi

**Affiliations:** 1 Department of HIV/TB Research & Training, HIV/TB Research & Training Center Bamako, USTTB; 2 Department of Public Health, Université de Montreal, Montreal, Canada; 3 Department of Global Health, Northwestern University, Chicago, USA; 4 Department of Neurology, Centre Hospital, University du Point «G», Bamako, Mali; 5 Department of Medicine, d’odontostomatologie, Bamako, Mali; 6 Department of Cellular & Molecular Pharmacology, Catholic University of Louvain, Brussels, Belgium; 7 Department of Infectious Diseases, Centre Hospital, University du Point «G», Bamako; 8 Department of Internal Medicine & Infectious Diseases, AIDS Reference Center, Catholic University of Louvain, Brussels, Belgium

**Keywords:** Antiretrovirals side effects, AZT, D4T, Mali, Proactive pharmacovigilance, Sub-Saharian Africa

## Abstract

**Background:**

There are few reports in the literature from sub-Saharan Africa (SSA) regarding antiretroviral-induced adverse drug reactions (ADRs). Antiretroviral therapy (ART) is now widely available in SSA, and ADRs during HIV infection are also frequent. In this study, we reported the frequency and risk factors of ART-induced ADRs in a Malian population.

**Methods:**

This prospective cohort study was performed in the HIV Care and Counseling Centre (CESAC) of Mali from 2011 to 2012. Adult patients infected with HIV and who had recently started ART were included and followed-up clinically Were included in this study, adult patients living with HIV and had recently started ART who were followed up for at least 6 months to determine the incidence of ADRs using Naranjo’s classification scale.

**Results:**

During this study, 357 (42.3%) patients presented ADRs (40.1% of our patients (n=338) experienced at least one ADR, and 2.2% (n=19) experienced at least two ADRs). The prevalence of ADRs by organ system was: 45.9% neurological (n=164); 29.4% metabolic (blood chemistry) (n=105); 15.4% hematological (n=55). High probable rate of ADR was observed as indicated by the Naranjo score in 83.7% of the cases. Zidovudine (AZT) and stavudine (d4T) use was identified as a risk factor for either anaemia or peripheral neuropathy whereas nevirapine (NVP) and female gender were risk factors for skin reactions. Patients with advance disease had the highest rate of ADRs compared to the others.

**Conclusions:**

Based on the Naranjo probability scale, our data show that ADRs such as peripheral neuropathy and anemia are very frequent. These ADR was linked to AZT and D4T. Our findings highlight the need for active monitoring, continuous pharmacovigilance of ART and change of some ART drug in this population.

## INTRODUCTION

Antiretroviral agents have brought hope for an extended lifespan to HIV-infected individuals in Mali as in worldwide. Unfortunately, undesirable adverse drug reaction (ADR) of antiretroviral therapy (ART) represent a real challenge in the management of HIV-infected patients especially in resource-limited setting. More than one quarter of HIV-infected patients do not continue the ART within the first year due to the adverse events ^[1]^ ADRs associated with ART affect numerous organs and have a variety of clinical presentations. The most prevalent side effects are dermatological and gastrointestinal (GI).^1^ ADRs to antiretroviral drugs in HIV-infected patients are a major cause for non-adherence and leading to treatment discontinuation, failure and the development of resistance to antiretroviral drugs.^2^ The Malian government has exerted continuous efforts to expand access to ART and also to provide new drugs with less toxicity. The National Committee against AIDS (HCNLS) established ART centres, to offer free HIV-treatment and opportunist infections (OIs).^3^ It is estimated that 37,000 Malian patients with HIV/AIDS needed ART in 2017.^3^ However, the Malian National Pharmacovigilance Program has only recently started operation in 2011. In Mali, there is a lack of well-trained health-care professionals who understand ART management and safety monitoring. Thus, many ADRs go unnoticed and unreported, and the implementation of ADR monitoring and reporting is therefore essential. The objective of this study is (i) to assess the nature, severity, and predictability of ADRs in Malian HIV-infected patients using ART, and (ii) to identify risk factors for ADRs in HIV-infected patients in Bamako, Mali.

## METHODS

The study was conducted at the HIV/AIDS Care and Counselling Centre (CESAC) after ethical approval of the study protocol by the IRB of the Faculty of Medicine, Pharmacy and Odonto-Stomatology at University of Bamako.

### Inclusion criteria

Patients were enrolled after signing the informed consent document, and all adult HIV-infected patients received a specific antiretroviral regimen for at least three months at the CESAC (843 were included in the study cohort).

### Exclusion criteria

Patients, who had received a previous round of ART and had interrupted the treatment for less than three months followed by a change of regimen, were not enrolled We did not enroll ART-naive patients, patients treated with ART for less than three months, or HIV-infected patients with acute or chronic co-morbidities such as tuberculosis (TB), viral hepatitis B or C, diabetes or cancer.

### An active pharmacovigilance method was adopted as

Between June 2011 and May 2012, a pharmacist monitored HIV patients intensively for any ADR. Visits to the ART Centre, were as follows: TO, the initial visit during which consent was obtained; T1, at two weeks after the start of ART; Ml, at one month post T1; M3, at three months post T1; M6, at six months post T1. We interviewed each patient and reviewed their medical charts at each follow-up visit for any signs of ADR. A senior clinical pharmacist assessed and discussed any suspected or documented ADR with the physicians’ team. Naranjo’s algorithm was used to assess the causality of each ADR.^4^ Moderate anemia was defined as hemoglobin (Hb) less than 10 g/dl; severe anemia: Hb < 7 g/dl. Lipodystrophy was established if changes in body fat distribution were reported by the patient and confirmed by the physician.

Metabolic side effects was defined using IDF 2005 criteria, which included waist circumference ≥94 cm in men or ≥80 cm in women, in addition to any two of the following four criteria.^5^ Triglycerides ≥1.50g/l, systolic blood pressure ≥130mmHg or diastolic blood pressure ≥85mmHg, HDL cholesterol <0.40g/l in men and 0.5g/l in women and glycaemia ≥1g/l. Orthers metabolics disorders specifically studied were diabetes (fasting glycose ≥1.26g/l), hypercholesterolaemia (Total cholesterol >2.2g/l). The patients divided into 4 groups according to the CD4 count (CD4GRP: 1 = CD4 <100; 2 = 100 <CD4 <350; 3 = CD4>350; 4 = CD4GRP4 = AIDS). We chose this classification to have homogeneous groups. All statistical analyses were performed using Statistical Package for Social Science (SPSS, Chicago, IL, USA) Version 17.0. A p-value <0.05 was considered statistically significant.

## RESULTS

During the two-year recruitment period, 5731 HIV-infected patients were enrolled and started ART (≥ 1 visit), of which 843 were included in the study cohort. Of these patients, 357 (42.3%) had adverse drug reaction (40.1% of our patients (n=338) experienced at least one ADR, and 2.2% (n=19) experienced at least two ADRs). Baseline characteristics of the patients are presented in Table 1.

Therapeutic regimens for 92.2% of patients included two nucleoside reverse-transcriptase inhibitors (NRTI) plus a non-nucleoside reverse-transcriptase inhibitor (NNRTI); 7.6% of patients were treated with two NRTIs and a protease inhibitor (PI), and 0.2% of patients were treated with three different NRTIs. The mean CD4 cell count (at baseline TO) was 204.8±16 cells/mm3. Due to the availability of drugs in Mali and the increasing incidence of AZT-induced anemia, stavudine (D4T) was used instead of zidovudine in some patients. The antiretroviral regimens used in patients and the Naranjo score are summarized in Table 2. The statistically significant difference between CD4 levels and the number (%) of Malian patients experiencing ADRs on ART is shown in Table 3.

**Figure 1:**
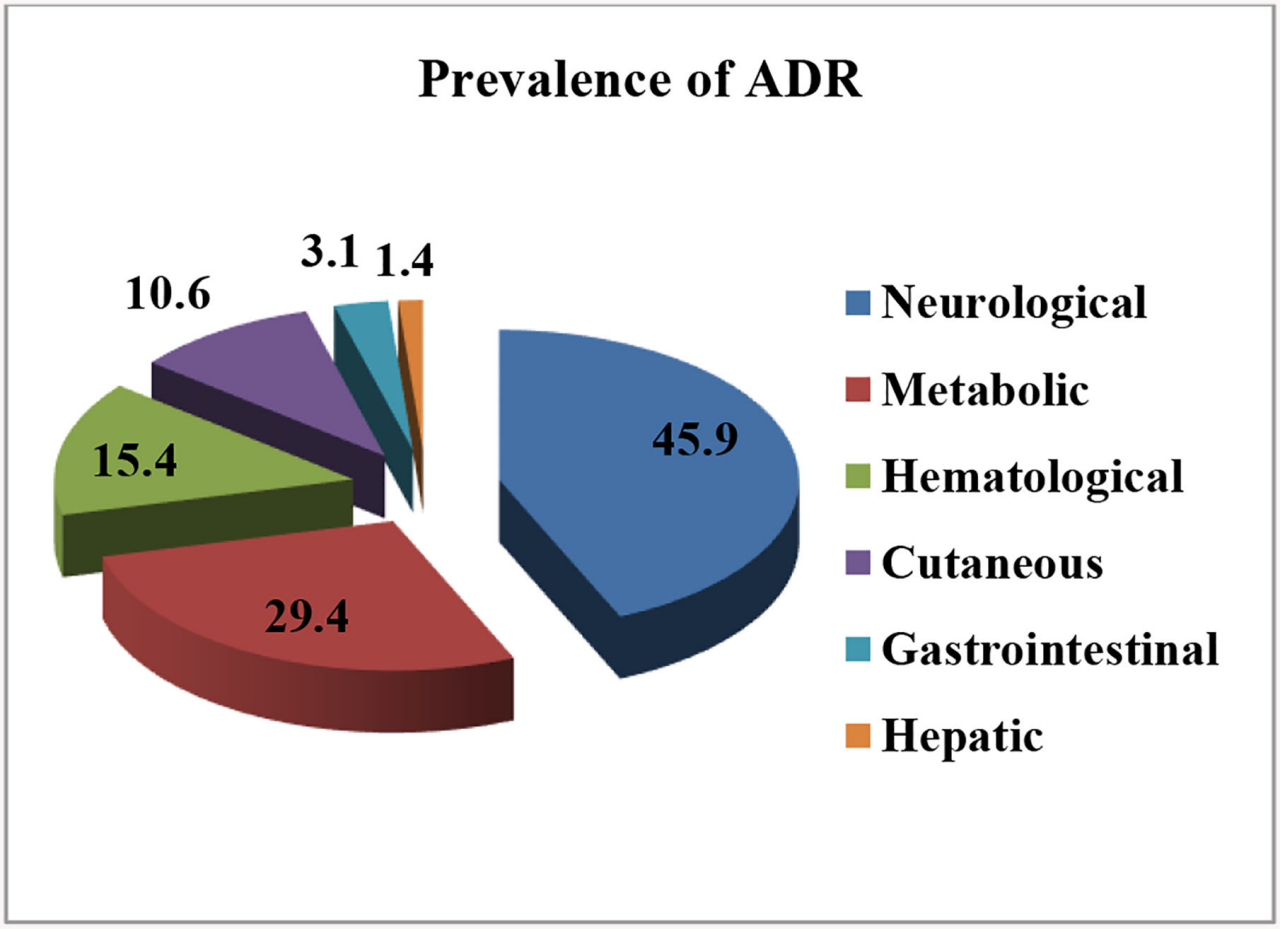
ADR by organ system.

**Figure 2:**
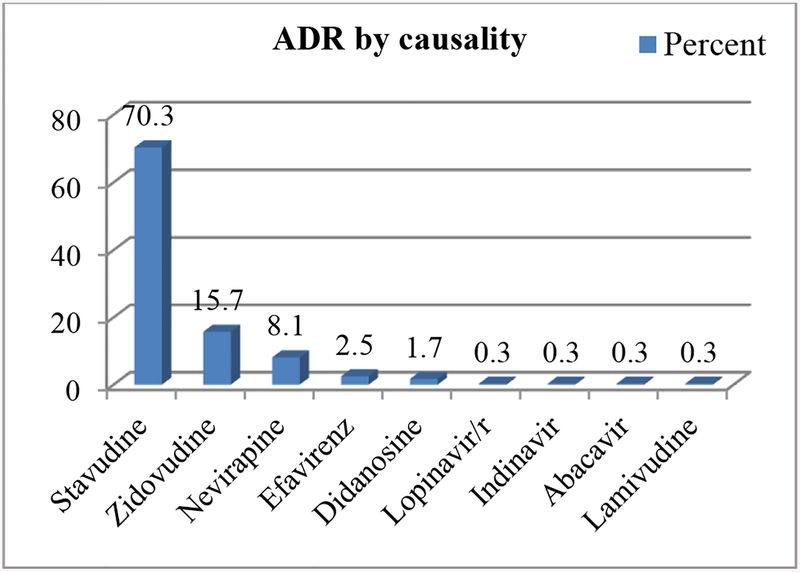
Incidence of ADR (%) according to antiretroviral drug.

The prevalence of ADR by organ system is summarized in Figure 1. Twenty-nine (29.4%) percent of patients experienced metabolic side effects. The incidences of ADR according to antiretroviral drug are summarized in Figure 2. D4T was associated with more adverse events (70.3%) than any other drug (Figure 2).

**Figure 3:**
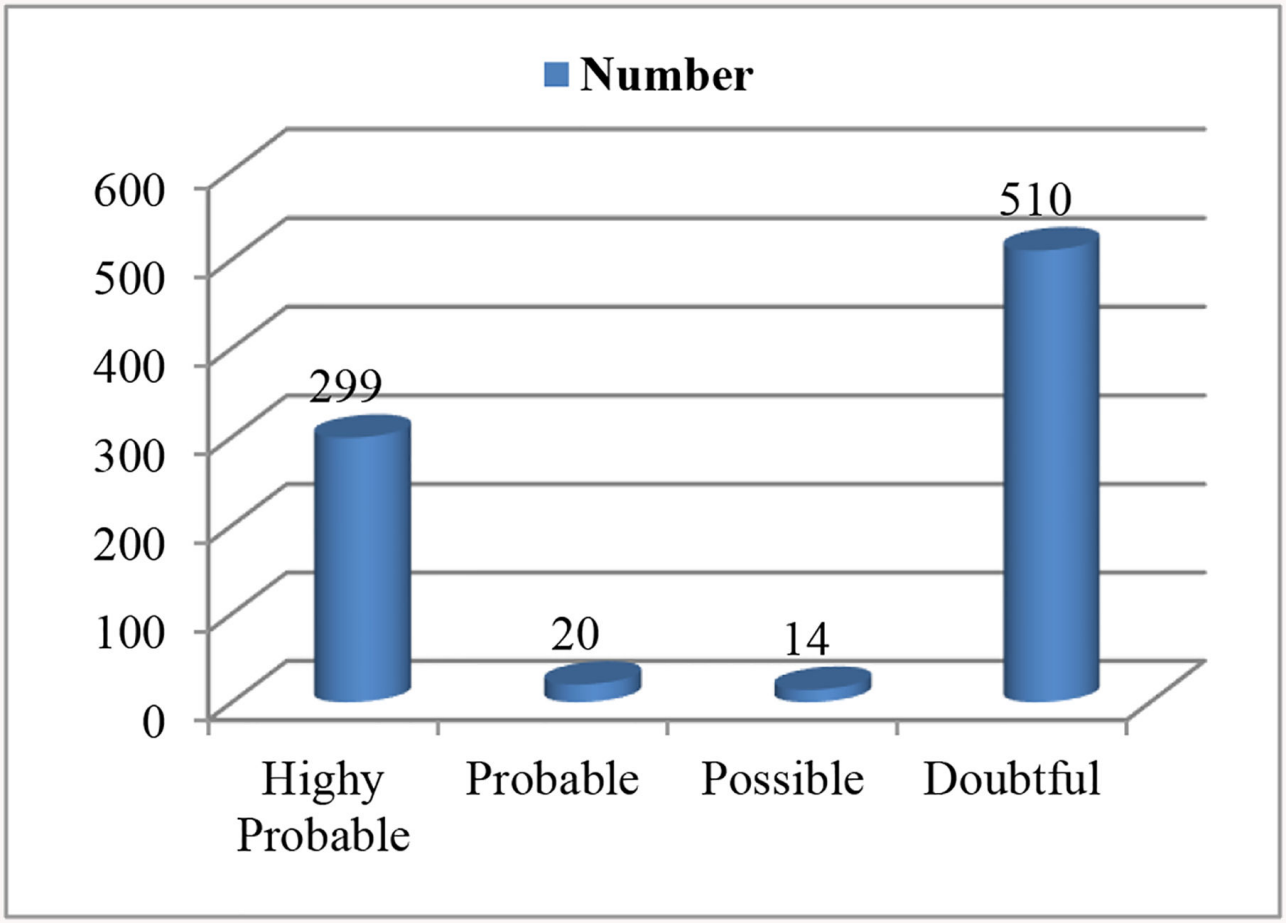
Adverse drug reactions using Naranjo’s score.

There was a high probability (35.4%) of adverse events based on the Naranjo score, the probability was 2.4%, the possibility of 1.6% and doubtful 60.6%. (Figure 3). Bivariate analysis identified CD4 count below 350 cells/ml and female gender to be risk factors for ADR to ART. Zidovudine therapy was found to be a risk factor for anemia, and D4Twas associated with a higher probability of peripheral neuropathy and lipodystrophy. On the other hand, the use of nevirapine (NVP) and female gender were associated with an increased risk of skin reactions and lipohypertrophy (Table 4).

Multivariate logistic regression analysis suggests that the regimens that did not include protease inhibitors (PI) resulted in fewer adverse events than the regimens that included a PI. Adverse events were positively associated with increasing age of patients. Patients with advance diseases (AIDS) had more adverse events compared to others (Table 5).

## DISCUSSION

This is the first study in Mali to evaluate the nature and severe morbidity associated with ADRs due to ARTs in HIV-positive adults with an active surveillance method. The data indicate no significant morbidity associated with ARTs in the local population. Gender-wise, the prevalence of ADRs observed was similar to that observed in other studies.^6^ The majority of ADRs observed in adults are similar to those seen in another study perform in South African hospital.^7^ Approximately 42.3% of patients showed ADR in the present study. This is a relatively low percentage, but it is considered accurate because of the large number of patients enrolled in this cohort.^8^

There is considerable data regarding the association between D4T and peripheral neuropathy, as well as the association between zidovudine and peripheral neuropathy.^9–11^ D4T is known to be a risk factor for peripheral neuropathy, we found that peripheral neuropathy was the most frequently reported ADR.^11^ Most of the patients who experienced peripheral neuropathy (88%) were treated with an ART regimen that included D4T for at least 6 months (OR: 12.7, P <0.05). In 55.4% (86/155) of these cases, D4T was discontinued. We found also few patients on zidovudine who had experienced peripheral neuropathy.

Metabolic effects were the second most frequently reported ADR in this study. It has been reported that NRTI and NNRTI drugs are not associated with significant metabolic side effects.^12^ After a year of treatment and follow-up of this cohort, approximately 12.3% of the HIV-infected patients experienced dyslipidemia and 2.9% experienced lipodystrophy, rejoins the results found in the study by Herman et al.^13^ Lipodystrophy cases in the literature have been described with several ART drugs but are usually associated NRTI.^14–16^ Our results confirm previous reports regarding metabolic side effects, although a greater proportion of our study participants used d4T.

HIV infection with or without AIDS can lead to anemia in 1.3 to 95% of patients.^17^ Among antiretroviral drugs, zidovudine is the only one known to cause reversible (upon discontinuation of AZT) myelosuppression in about 24% of patients.^17,18^ We noted hematological ADRs in patients with AZT-containing regimens in 15.4% of cases. Anemia and other hematological ADRs were the most in patients co-infected with HCV and/or HBV.^19^ Anemia occurred in patients receiving zidovudine-containing regimens within the first few weeks or months after initiation of therapy. Similar to the findings of Koduri et al we did not observe many cases of severe anaemia and hemoglobin levels increased after discontinuation of zidovudine.^18^ Huffam et al, found that young female HIV patients with low baseline hemoglobin (Hb <10 g/dl) were at higher risk of zidovudine-induced anemia.^20^ A highly significant association (OR: 61.65, P <0.0001) was also revealed between the use of zidovudine and the occurrence of anemia, as previously reported.^17^ However, in this study, young age and female gender were not significantly associated with anemia. In this study, we only considered treatment with zidovudine for patients with a baseline Hb level above 8 g/dl.

In this study, Skin reactions were not the most prevalent and were observed in only 7.8% of the patients enrolled. This may be due to the World Health Organization’s protocol used in Mali, which recommends an initial titration of NVP to avoid ADRs. Among the ART-induced skin reactions, mild to moderate maculopapular rash was the most frequent and was most often reported by patients treated with regimens containing NVP and/or efavirenz (EFV).^21^ ART-induced skin rash is highly prevalent in female HIV-positive patients with high CD4 counts (above 250 cells/mm^3^).^22^ NVP use (OR: 1.13) (p=l) and female gender (OR: 1.87) (p=0.129) are risk factors for skin reactions under intensive monitoring.^23^ AZT-induced skin reactions resulted in drug discontinuation in 8% of our cohort, but skin discoloration was rare.^8^ ART-induced severe hepatotoxicity was observed in 1.4 *%* of the patients followed in this cohort compared to 2 to 18% reported in the literature^[24]^. The incidence is even higher among HCV and/or HBV co-infected individuals.^19^ GI adverse effects were reported more frequently in ART regimens containing Pis and/or NRTIs.^25^ We recorded few cases of mild GI complaints, and these generally resolved with symptomatic treatment.^26^

HIV infection itself or ART (tenofovir, and indinavir) toxicity has been incriminated in acute or chronic HIV-related renal impairment.^27–29^ Therefore, the observed increase in azotemia in some of our patients may be related to their hydration status.

While spontaneous notification of ADR, dosing modification, or interruption is more suitable for the detection of late drug-related adverse events, intensive monitoring allows for the detection of ADR early in the context of ART scale-up in Africa.^30,31^

## CONCLUSION

In this prospective study, we investigated antiretroviral-induced ADRs in adult HIV-infected patients in Mali. Our results showed ART with D4T, AZT, and NVP is associated with a number of ADRs in this Malian population, similar to those seen in other populations. Female HIV-infected patients with CD4 <350 cells/μl have high risk for skin reactions and require intensive monitoring. Attention must be focused to the monitoring of ADRs associated with antiretroviral medications while simultaneously improving access to ART for HIV infected people in Mali.

## Figures and Tables

**Table 1:** Patient demographics.

Variable	N	843
Age	Mean±SD	36.9±10.1
	Median (P25, P75)	36.0 (29.0, 44.0)
	Min, Max	(18,76)
CD4 level	Mean±SD	204.8±164.1
	Median (P25, P75)	181 (72, 305)
	Min, Max	(1, 967)
	Missing Value	68 (8.1%)
Gender	Male	237 (28.1)
	Female	606 (71.9%)
Occupation	State worker	175 (20.8%)
	Trader	166(19.7%)
	Homemaker	221 (26.2%)
	Unemployed	106 (12.6%)
	Other	175 (20.8%)
Therapeutic regimen	2NRTI + NNRTI	777
	2NRTI + PI	64
	3NRTI	2

**Table 2:** Proportion of subjects experiencing side effects according to treatment and Naranjo scale.

Number of drugs	Naranjo Scale				
	NA	Doubtful (N, %)	Possible (N, %)	Probable (N, %)	Highly probable (N, %)
1	NA	234 (27.76)	13 (1.54)	9 (1.07)	9 (1.07)
2	326 (38.67)	55 (6.52)	7 (0.83)	5 (6.59)	13 (1.54)
3	119 (14.12)	3 (0.36)	NA	NA	2 (0.24)
4	41(4.86	3 (0.36)	NA	NA	
5	NA	4 (0.47)	NA	NA	

**Table 3:** Number (%) of Malian patients experiencing adverse drug reaction on ART according to CD4 count.

Number of AE	CD4 count			
	<100	100–350	>350	AIDS
0 (N, %)	155 (18.39)	239 (28.35)	7.4 (8.87)	18 (2.14)
1 (N, %)	62 (7.35)	158 (18.74)	36 (4.27)	43 (5.10)
2 (N, %)	9 (1.07)	7 (0,83)	2 (0.24)	2 (0.24)
3 (N, %)	6 (0.71)	4 (0.47)	1 (0.120	3 (0.36)
4 (N, %)	8 (0.95)	10 (0.19)	4 (0.47)	2 (0.24)

P-value of Cochrane-Mantel-Haenszel Statistic P<0.0001

**Table 4:** Bi-variate analysis of risk for adverse drug reactions to antiretroviral therapy in intensively monitored patients.

Variable	Value	Odds Ratio (95% CI)*	P-value
Age (years)	<20	1 (reference)	
	21–40	0.66 (0.22–1.74)	0.676
	41–60	1.81 (0.67–5.86)	0.262
Gender	Male	1 (reference)	
	female	1.76 (0.56–8.04)	0.455
CD4 Count (Cells/μl)	>350	1 (reference)	
	<350	0.29 (0.09–1.02)	0.075
Number of ADR	One	1 (reference)	
	Two	2.07 (0.28–8.11)	0.390
Peripheral neuropathy	stavudine −	1 (reference)	
	stavudine +	12.78 (6.28–29.83)	<0.001
lipoatrophy	stavudine −	1 (reference)	
	stavudine +	11.32 (4.07–48.79)	<0.001
Lipohypertrophy	nevirapine −	1(reference)	
	nevirapine +	29.28 (15.66–54.76)	<0.001

**Table 5:** Regression analysis of ADR risk factors.

Odds Ratio Estimates			
Effect	OR	95% Wald confidence interval	
Age	1.016	1.001	1.031
Gender (M/F)	1.559	1.115	2.181
Therapeutic regimen (IP− vs IP+)	0.531	0.296	0.953
CD4GRP* 1 vs 4	0.188	0.102	0.346
CD4GRP* 2 vs 4	0.246	0.137	0.441
CD4GRP* 3 vs 4	0.212	0.108	0.415

* CD4GRP: 1 = CD4 <100; 2 = 100 <CD4 <350; 3 = CD4>350; 4 = CD4 = AIDS
